# Supplementary material for: Predicting natural variation in the yeast phenotypic landscape with machine learning
Source: Mol Syst Biol. 2025 Sep 1;21(11):1466–89. doi: 10.1038/s44320-025-00136-y (PMC12583546; doi:10.1038/s44320-025-00136-y)
Supplement: Supplementary file 1 — Appendix [file 44320_2025_136_MOESM1_ESM.pdf]

Appendix for “Predicting natural variation in the yeast phenotypic landscape with machine learning”

Sakshi Khaiwal[1,#], Matteo De Chiara[1], Benjamin P Barré[1], Inigo Barrio-Hernandez[2], Simon Stenberg[3], Pedro Beltrao[2], Jonas Warringer[3] and Gianni Liti[1,#]

[1]CNRS, INSERM, IRCAN, Côte d'Azur University, Nice, France; [2]Institute of Molecular Systems Biology, ETH Zürich, 8093, Zürich, Switzerland; [3]Department of Chemistry and Molecular Biology, University of Gothenburg, 40530 Gothenburg, Sweden.

# to whom correspondence should be addressed: S.K. sakshikhaiwal@gmail.com; G.L. gianni.liti@cnrs.fr

Table of contents:

Appendix Note S1. Impact of population structure on prediction……………………………………...…3

Appendix Note S2. Multi-target regression models accurately predict the compound phenome…..……4

Appendix Figure S1. Phenotype anticorrelation networks and clustering……………..…………………..5

Appendix Figure S2. Phenotype correlations…………………………………………………………6

Appendix Figure S3. GWAS hits analysis and statistics………………………………………………7

Appendix Figure S4. Pleiotropic genes with GWAS hits……………………………………………..8

Appendix Figure S5. GWAS network expansion……………………………………………………...…9

Appendix Figure S6. Feature selection and model benchmarking………………………………..…10

Appendix Figure S7. Traits predictability…………………………………………………………11

Appendix Figure S8. Benchmarking predictors.……………………………………...……….…..12

Appendix Figure S9. Average FI scores distributions.………………………………...…………..13

Appendix Figure S10. Functional analysis of impactful features………………………...……...…..14

Appendix Figure S11. Multiple phenotypes correction for GWAS using FDR correction...……..……15

Appendix Figure S12. Multi-target prediction.………………………………...…………………16

Appendix Figure S13. Entire phenome predictions.……………………………...…………………17

Appendix Figure S14. Phenome prediction by clades and phenotype classes…………………………18

Appendix Figure S15. Prediction accuracy vs genetic distances………………………………...…..19

Appendix Table S1. Comparing feature selection methods.………………………..……………….20

Appendix Note S1

Impact of population structure on prediction

The *S. cerevisiae* has a fairly strong population structure, and the collection is divided into 30 major clades(Peter *et al*, 2018). This can represent a confounding factor and be treated as having real biological effects by ML models(Whalen *et al*, 2022). To reduce this bias, we measured the population structure and added it as an input feature for all predictions shown. Furthermore, to quantify the impact of the population, we tested the predictions derived from three splitting criteria: 1) Hold-out at random (HOAR), 2) Intra-clade hold out (INHO), and 3) LOCO (leave-one clade out). HOAR includes strains from all the clades (1,011 strains) and randomly divides them into a 75% training set and a 25% testing set. The INHO strategy considers only the Wine European clade (WE) with 276 strains which have a genome-wide SNP difference of only 0.12% (compared to an average of 0.5% overall) and a relatively weak internal population structure(Peter *et al*, 2018), so both the training and testing samples come from almost an independent and identically distributed set. The strains are randomly divided into a testing set with 50 strains and the rest for training to maintain a sufficient number of strains. Finally, LOCO consists of using a complete clade (in this case, 'M3.Mosaic_Region_3') as the test clade and using the rest of the clades for training. In this case, the training and testing samples most likely have distributional differences arising due to the inherent biological structure. We performed the predictions for the 30 test phenotypes using ‘BayesGBM’ for all three strategies (Appendix Fig. S8C). HOAR gave significant predictions for almost all phenotypes, while INHO and LOCO gave significant predictions for only 50% of the phenotypes. However, the average accuracy reduced significantly for INHO and LOCO. The lower number of phenotypes predicted with INHO could be due to the significant decrease in the training samples (almost by 1/4th) compared to HOAR. Moreover, the high genetic distance between the strains in the training and the test set could explain the reduced performance using the LOCO strategy. This suggests that predictions depend highly on the phenotypes and are predicted well if the relation between the genetic and phenotypic variation is generalized over the entire population (i.e., it is the same for all subclades). We also tried different sizes of training and test sets (Appendix Fig. S8D). While no comparable difference was seen across the splitting percentages between the train and test set, there was a significant variation among different folds for the same training, suggesting the composition of the training set can have a considerable impact on the model performance.

Appendix Note S2

Multi-target regression models accurately predict the compound phenome

To go beyond single-target predictions, we used two multi-target regression methods to construct models that can predict the 223 phenotypes at once (Appendix Fig. S12A). We used the PA, P(AF) scores, and LOF as they were the best predictors across the 30 phenotypes test. We randomly divide data into a training (75%) and a testing (25%) set and run predictions using multitask LASSO, gradient-boosting decision trees, and a deep neural network. The overall average accuracy remained around 0.4 irrespective of the methods and input predictors (Appendix Fig. S13). Despite being linear, Multitask LASSO performed comparable to the multi-regressor GBMs and deep neural network (Appendix Fig. S12A-C). Moreover, the PA gave the best results among the three predictors (Appendix Fig. S13A, D-E).

Next, we defined strain-level accuracy as all the phenotypes predicted vs. measured for each test strain and phenotype-level accuracy as all values predicted vs. measured for each phenotype considering all test strains. We observed significant variation in strain-level accuracy across clades, not depending on the clade's size (Appendix Fig. S12B and Appendix Fig. S14A). Instead, accuracy per clade negatively correlated with the average genetic variation (number of ORFs and SNPs differences) per clade (Appendix Fig. S15). This suggests the strains that are genetically similar to each other are predicted better compared to the distant strains. We also observed significant phenotype-level accuracy variation ranging from 0 to 0.75 (Appendix Fig. S14B). Growth in nitrogen and sugar sources have the largest number of inaccurate predictions, consistent with results from individual phenotypes (Appendix Fig. S14B). We extracted the feature importance for P(AF) scores as predictors from LASSO and GBMs. In both cases, *SFL1* was reported among the highest scored genes, which is also a top hit from the GWAS impacting many phenotypes. Furthermore, the top-scored genes from both cases are enriched in a number of important processes such as binding, cellular, and metabolic processes (Dataset EV8).

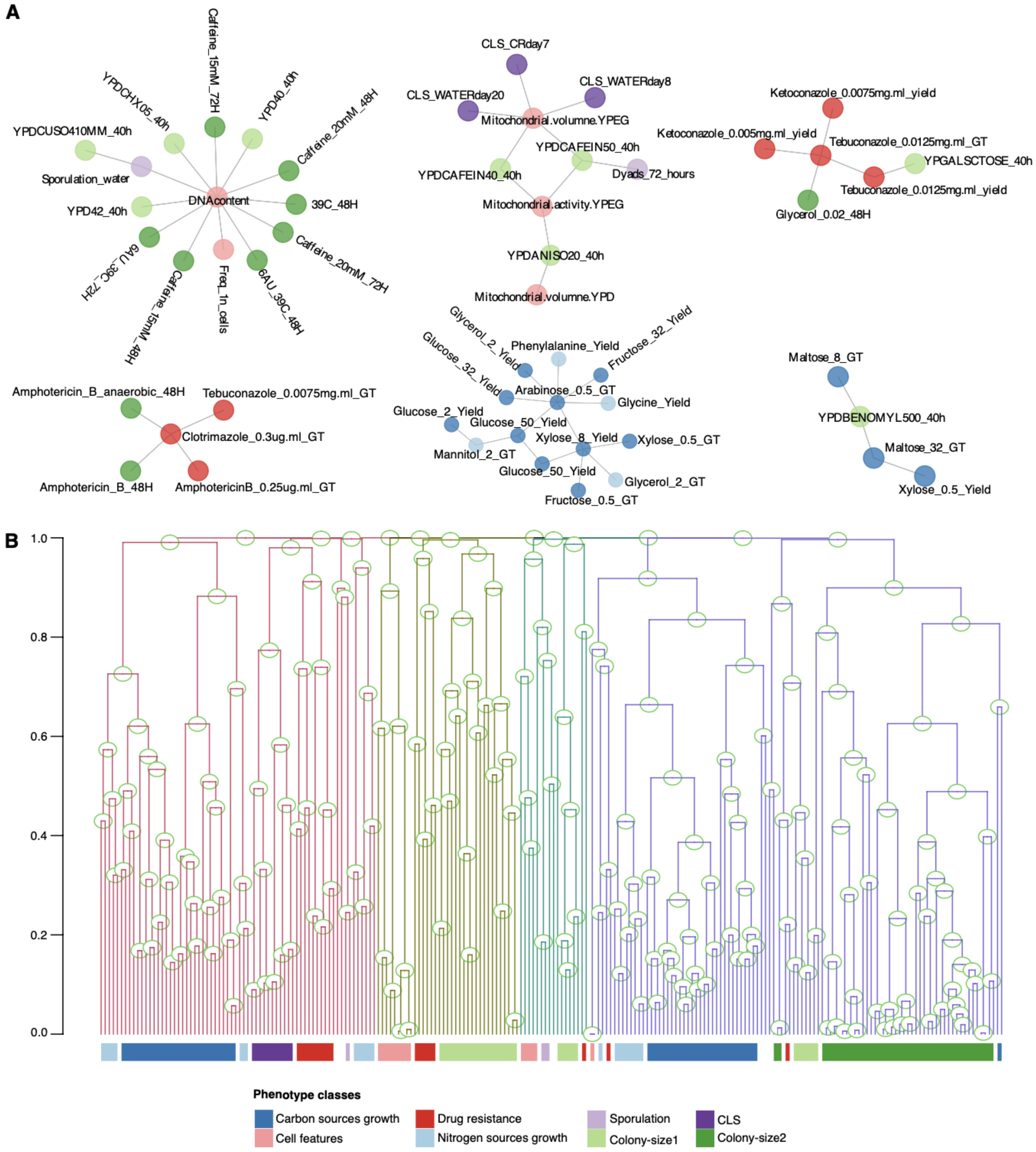

Appendix Figure S1 | Phenotype anticorrelation networks and clustering. A, Anticorrelations produced six clusters (with Pearson's coefficient <= -0.3) mainly consisting of trade-offs between growth rate and yield in various conditions. **B,** Hierarchical clustering groups phenotypes into four major groups (line colours) that are largely consistent with correlation patterns (Fig. 1B) and phenotypic classes (bottom coloured bar).

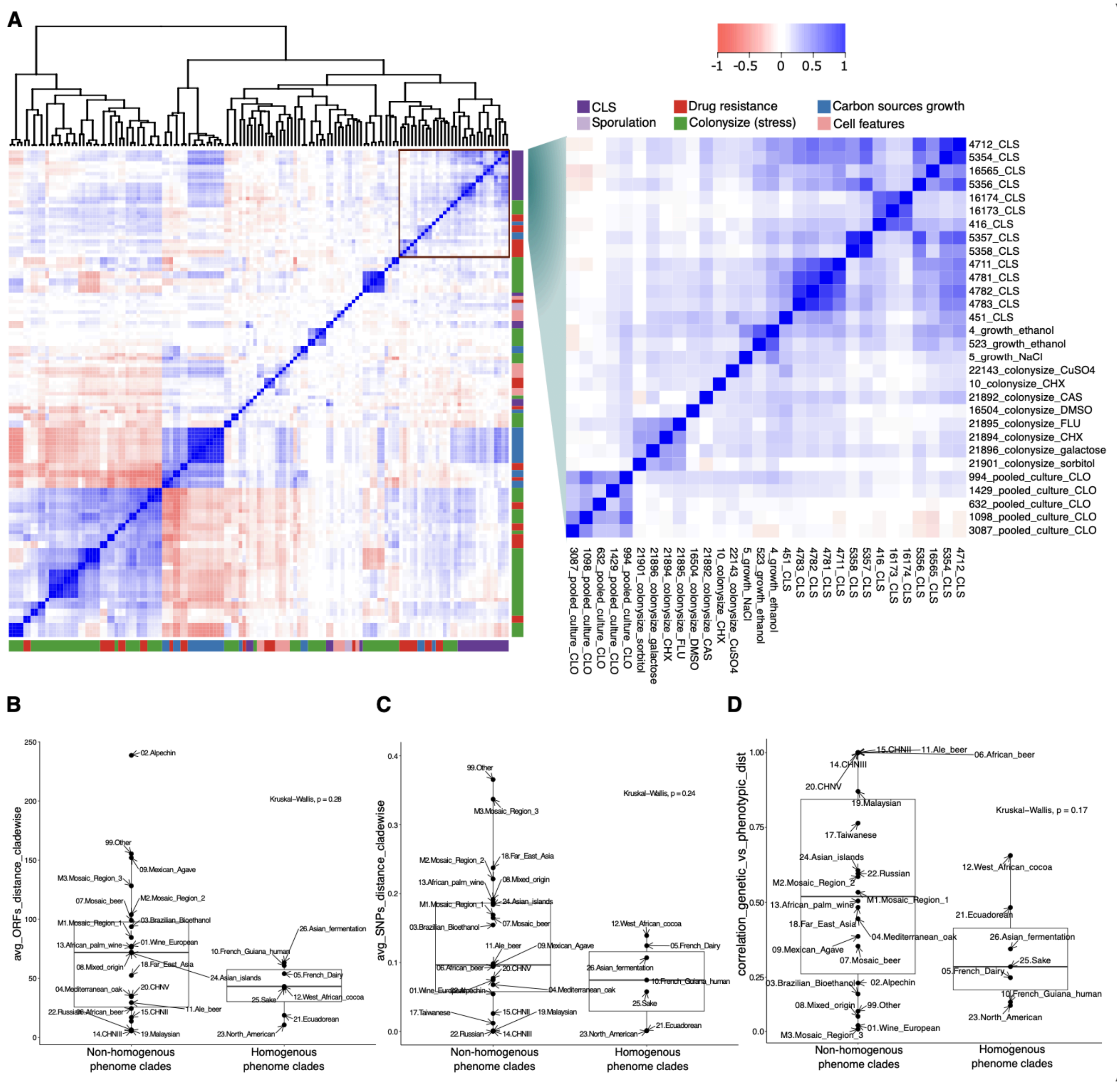

Appendix Figure S2 | Phenotype correlations. A, Phenotype similarity matrix extracted for the gene-knockout phenotypes confirms similar patterns of correlations, including the antifungal drug resistance and CLS (right zoom-in). **B-D,** comparison between the average genetic variation between the clades highlighted in Fig. 1C and partitioned by homogenous (clades with strains clustered together based on their phenotypic distances) vs non-homogenous phenome (rest of the clades). **B,** The average SNPs difference between strains for each clade is lower for clades having a homogenous phenome’ compared to the other clades, except for clades with few strains. **C,** Average number of ORFs differences similarly showed lower variation for the clades with homogenous phenome. **D,** Correlation between genetic variation vs phenotypic variation also showed a similar trend.

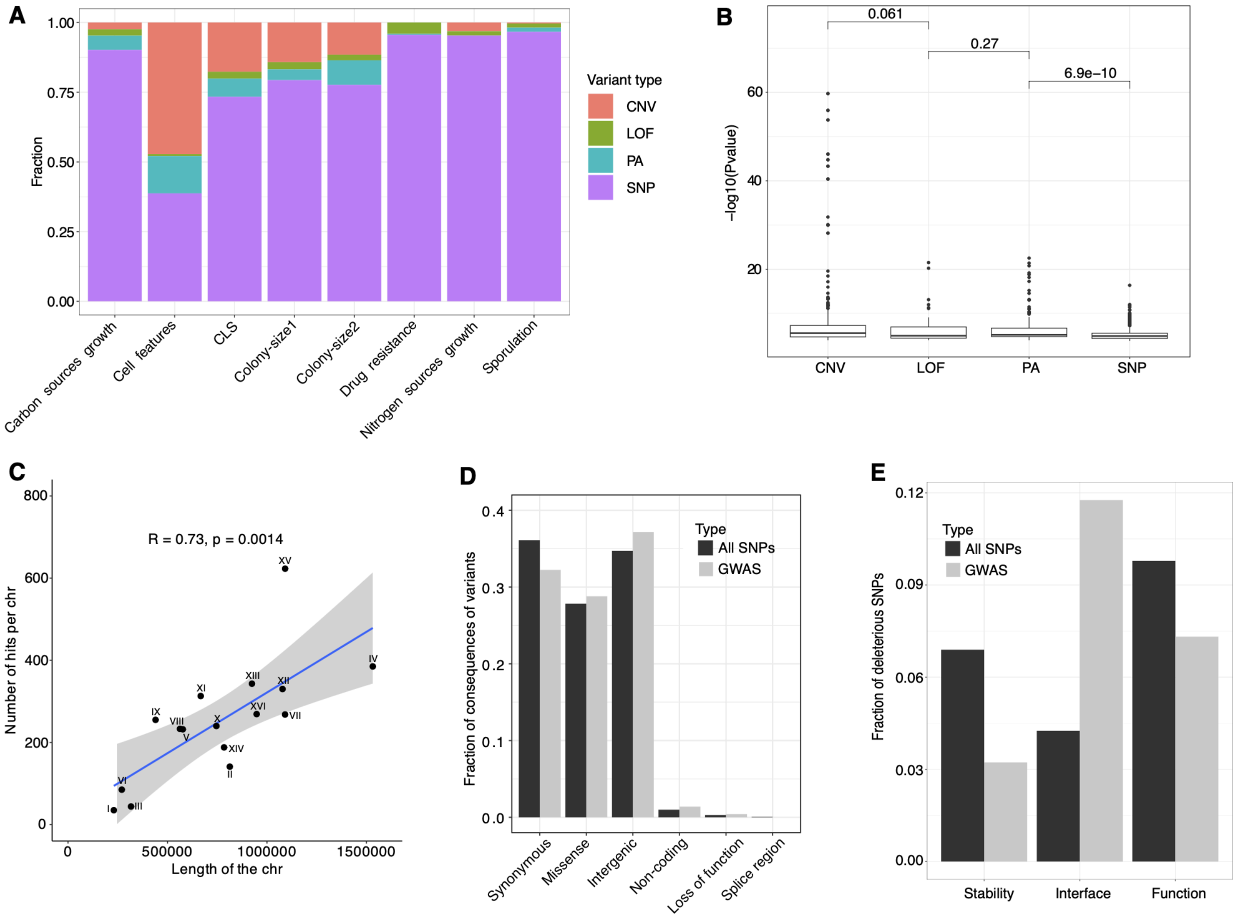

Appendix Figure S3 | GWAS hits analysis and statistics. A, Fraction of various types of significant GWAS determinants in different phenotype classes. **B,** Comparison between the distributions of strength of associations between different types of genetic determinants (Wilcoxon test). **C,** The number of GWAS hits obtained for the entire phenome per chromosome shows a significant correlation with chromosome lengths, except for chromosome XV which has an exceptionally high number of hits. **D,** The number of variants in each category of the mutations obtained from the set of SNPs of the population reflects the proportion of variants obtained as hits from GWAS, except for variants in splice regions, however, this could be due to their small sample size (280 in the entire population). **E,** The fraction of deleterious SNPs in the SNPs set vs the GWAS hits shows that a higher number of SNPs impact the protein function compared to the stability of the protein. Although the number of deleterious SNPs affecting the protein-protein interface seems to be enriched in the GWAS, this is due to the very low number of predictions available for the interface interactions from mutfunc(Wagih *et al*, 2018) (Dataset EV4).

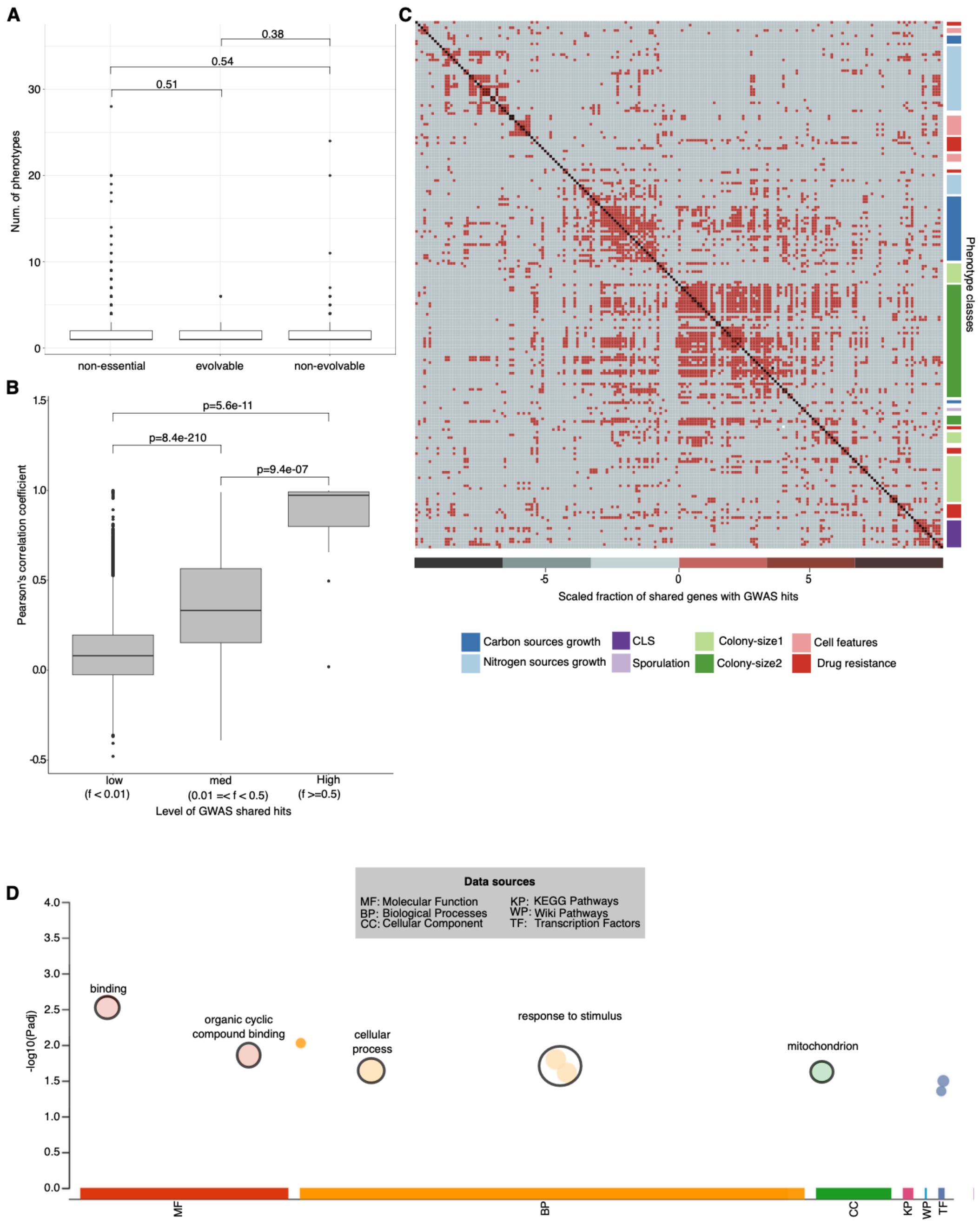

Appendix Figure S4 | Pleiotropic genes with GWAS hits. A, The distributions of the number of phenotypes impacted per gene show no significant differences between the essential (evolvable and non-evolvable) and non-essential gene categories(Liu *et al*, 2015). **B,** We divided all pairs of phenotypes into three categories: low (<0.01), medium (0.01-0.5), and high (>0.5) fraction of shared GWAS hits. The fraction of GWAS hits shared between each pair of phenotypes is directly proportional to the level of correlation between the two. **C,** GWAS associations sharedness among phenotypes at the gene level (considering cumulative hits in a given gene). The number of shared genes with GWAS hits between each phenotype pair (heat map) reflects the pattern of the phenotypic correlation map (as visible from the right sidebar of the phenotypic classes). **D,** The pleiotropic genes (impacting more than 1 phenotype) from GWAS show enrichment in processes such as binding, organic cyclic compound binding, cellular response to stimulus.

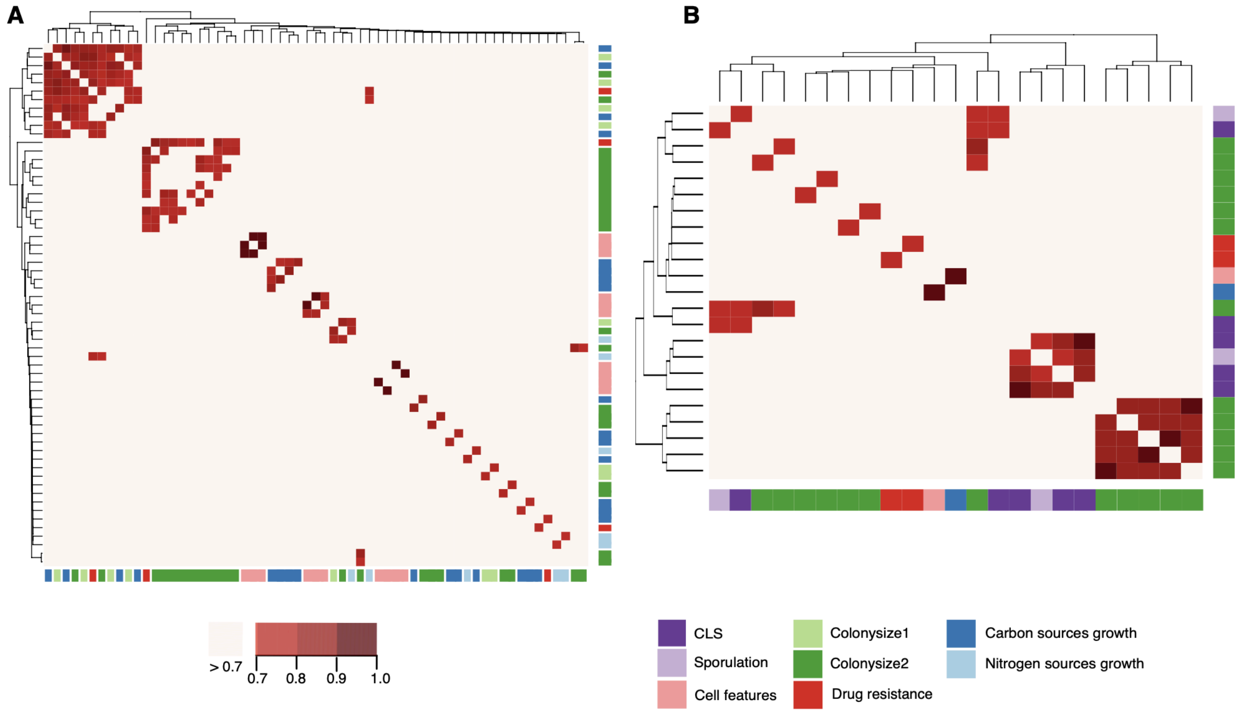

Appendix Figure S5 | GWAS network expansion. A-B Network expansion analysis for the natural population and the gene-deletion collection respectively. The heatmap shows the percentage of overlapping genes between the gene modules associated with each phenotype. Only the phenotype pairs with 75% or higher number of overlapping genes are shown in red. **A,** Most phenotype pairs with a high number of overlapping modules are between phenotypes associated with similar conditions (along the diagonal) except the cluster on the top left position which consists of growth phenotypes measured in several carbon-rich (maltose, sorbitol, and galactose) and stress (caffeine, paraquat, and tebuconazole) conditions. **B,** Similar patterns emerge in the gene-deletion collection with most overlapping modules between similar traits and conditions. The two big clusters on the bottom right corner are among CLS and colony-size in stress conditions respectively.

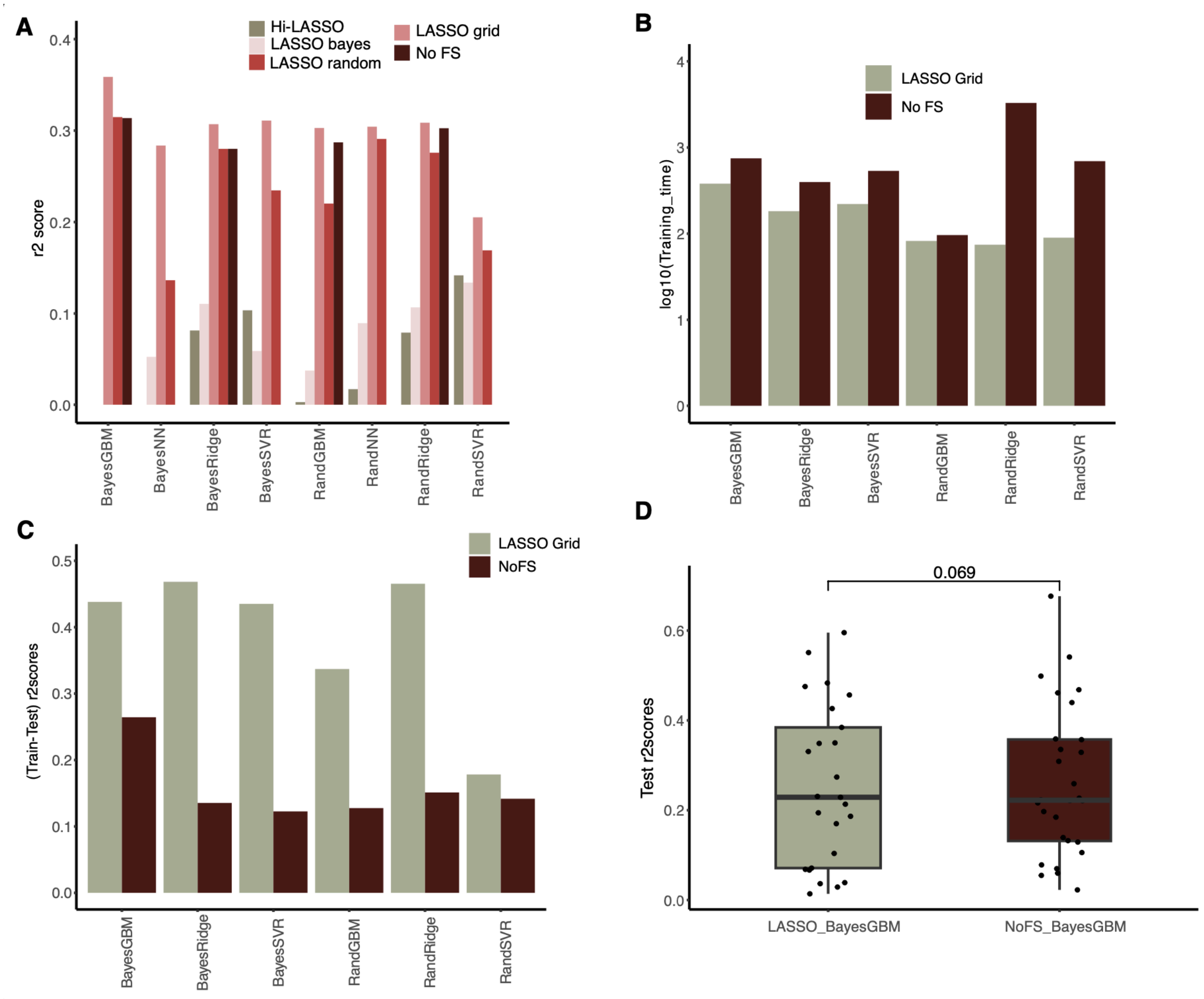

Appendix Figure S6 | Feature selection and model benchmarking. A, Comparing prediction models with different LASSO regression models (grid LASSO, random LASSO, Bayesian LASSO and Hi-LASSO) and without feature selection **B,** The training times are reduced by a significant fraction for most of the methods when the number of features is reduced using an a-prior feature selection making the training process efficient. **C,** All models exhibited a greater difference between the training and testing sets when feature selection was applied, indicating that they performed significantly better on the training set than on the test set. **D,** No significant difference between the accuracies was observed between predictions when LASSO feature selection vs no pre-feature selection, based on a subset of 30 phenotypes (paired Wilcoxon test). .

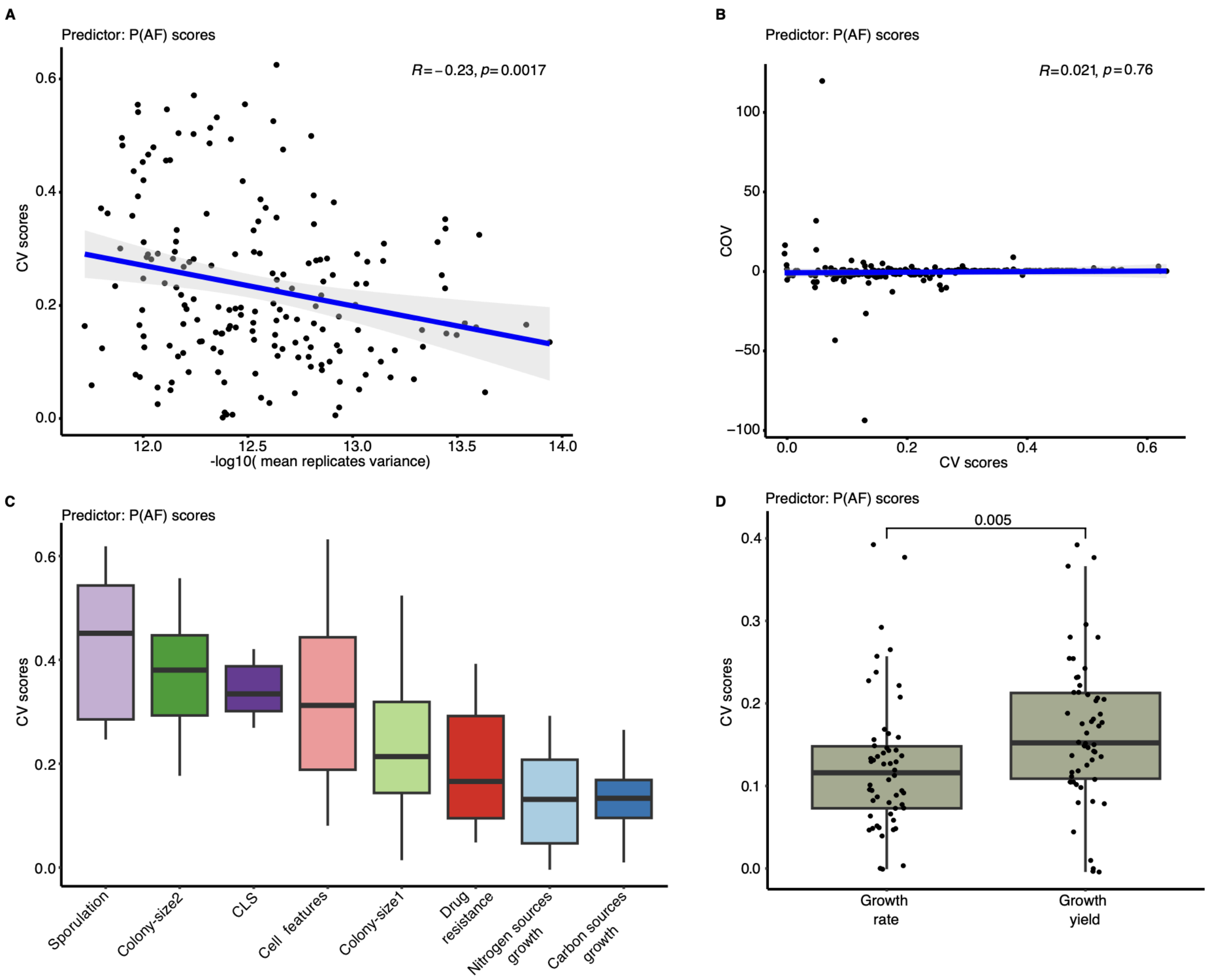

Appendix Figure S7 | Traits predictability. The mean of the five-fold cross-validation r2 scores (CV scores) from the predictions was used for comparisons. **A,** The CV scores from phenotypic predictions are significantly negatively correlated with the variation in the measurement to a small extent. No correlation was detected between the coefficient of variation (COV), which depicts phenotypic variability, and the predictability of the phenotype. **C,** The variation in the predictability of the phenotype among different phenotype classes. **D,** Growth yield shows significantly better predictions compared to the growth rate measured in identical conditions (Wilcoxon test).

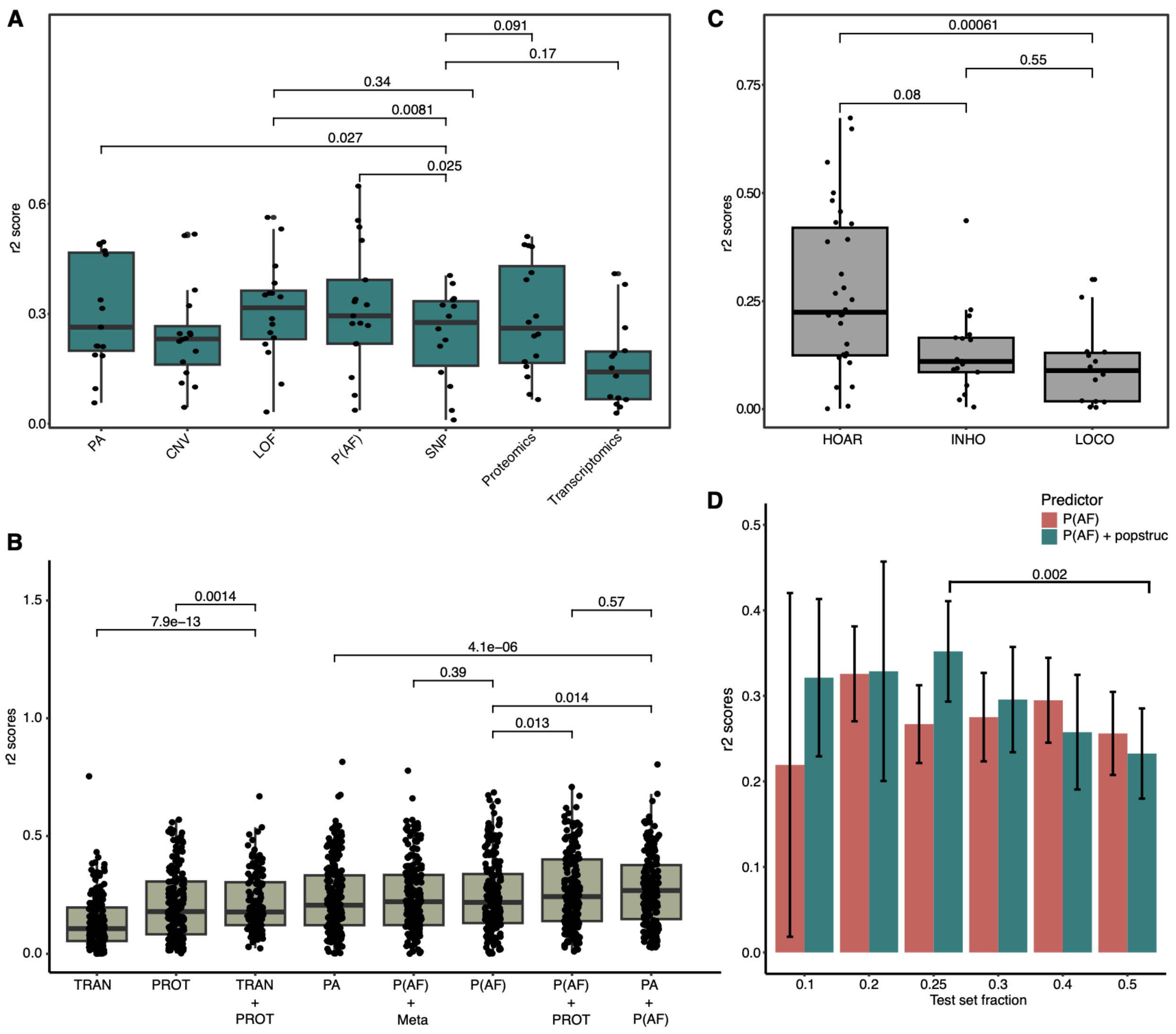

Appendix Figure S8 | Benchmarking predictors. A, The predictions for ~20 random phenotypes based on different predictors (PA, CNV, P(AF), LOF, SNP, transcriptomics, and proteomics) showing that the predictions from SNPs are comparable to the other predictors. **B,** Comparison between different predictor combinations shows that the accuracy is rescued when combining proteomics with transcriptomics. **C,** Predictions for the three splitting strategies i.e., hold-out at random (HOAR), intra-clade hold out (INHO) and leaving-one clade out (LOCO) tested for 30 phenotypes. However, the number of meaningful predictions (r2>0.0) significantly reduced from ~95% for HOAR, to ~50% for INHO and LOCO. **D,** Comparison between different train and test sample sizes with P(AF) scores and P(AF) scores with population structure as predictors over 5 iterations (Wilcoxon test). Test set shows the fraction of data excluded for the test set.

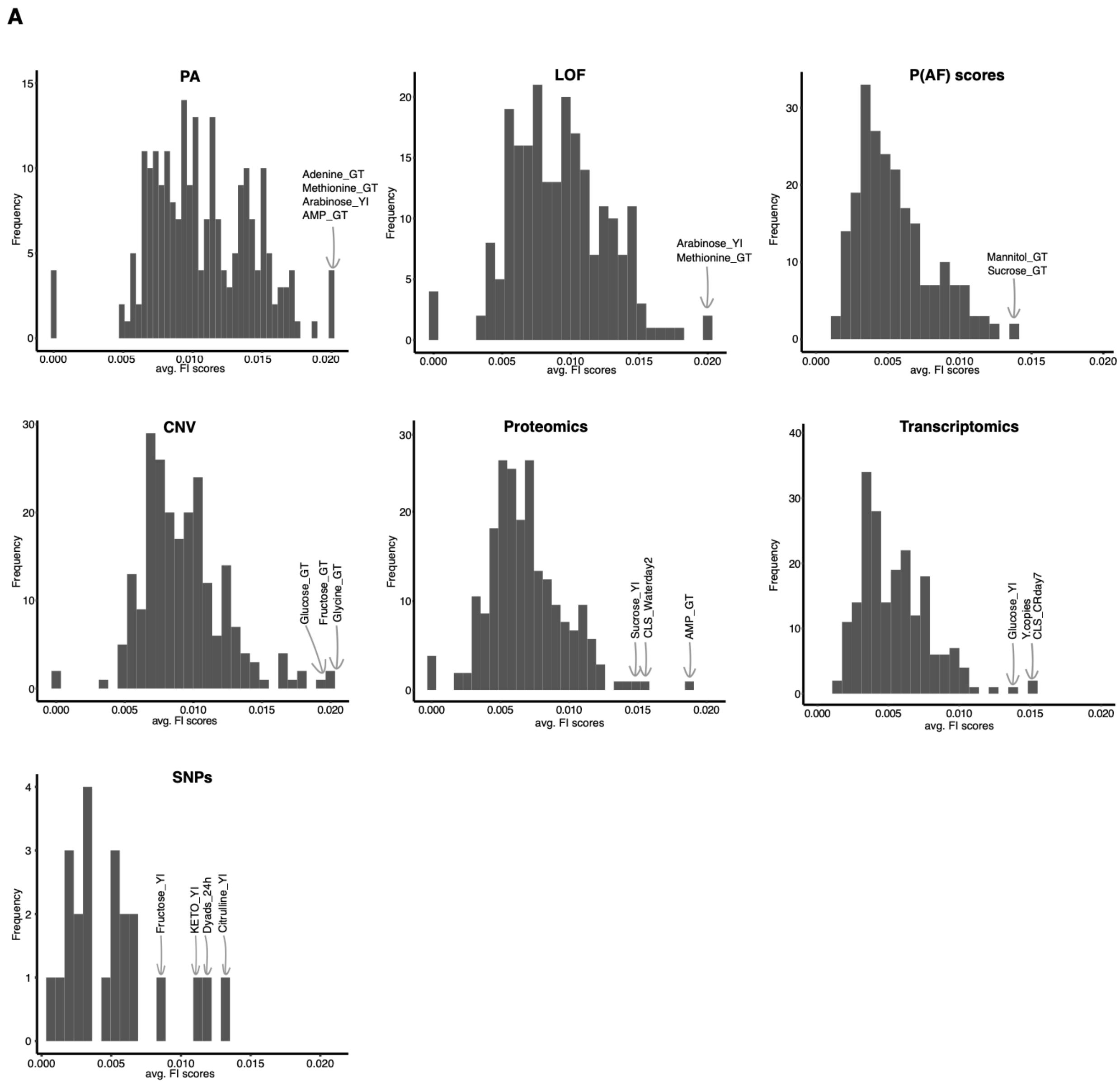

Appendix Figure S9 | Average FI scores distributions. Panels (A) - (G) show the distribution of average top 50 features FI scores per phenotype for each predictor, highlighting the top average phenotypes for each case.

A

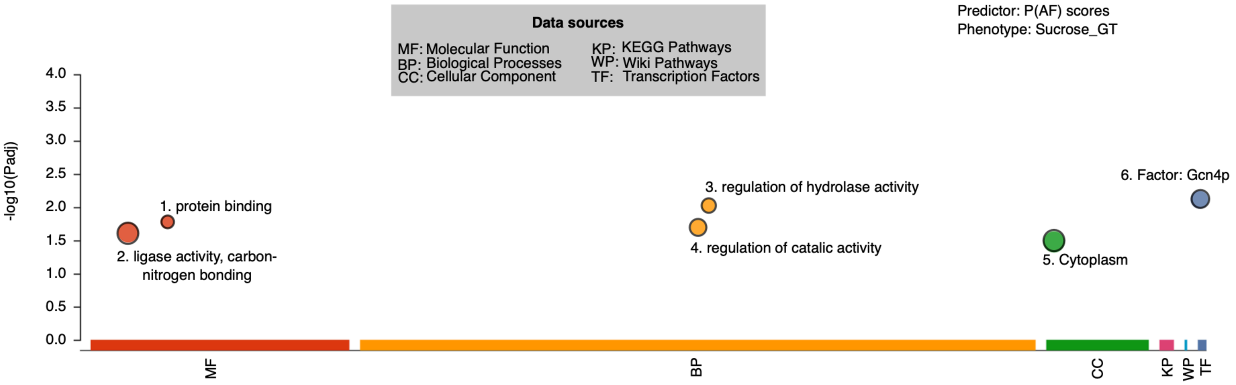

B

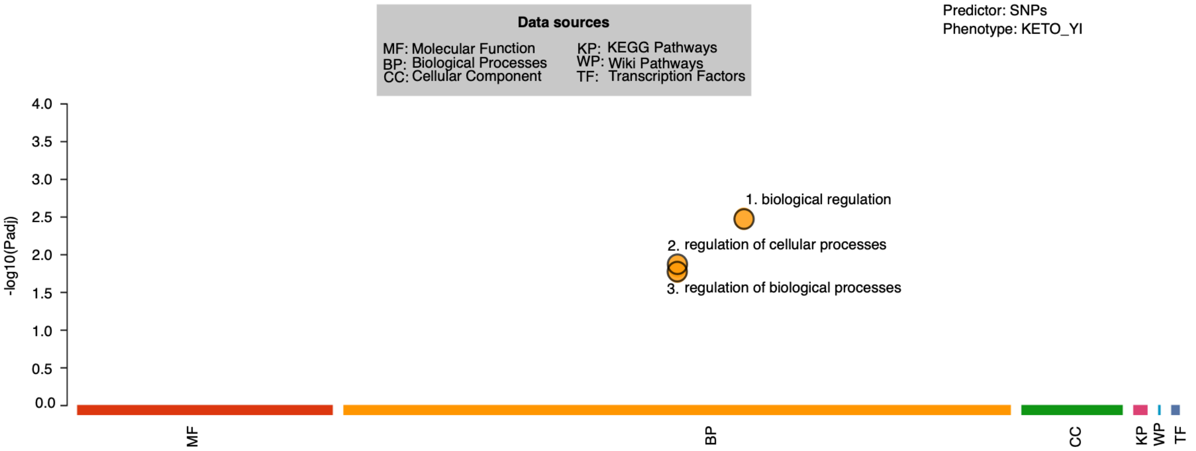

Appendix Figure S10 | Functional analysis of impactful features. Panels (A) and (B) show respectively the go-enrichment results from gprofiler (https://biit.cs.ut.ee/gprofiler/gost) for two of the top phenotypes, growth measured in sucrose and ketoconazole respectively. **A,** The genes with FI score > 0.01 for growth rate in sucrose show enrichment in protein binding, ligase activity, ect. **B,** The genes with FI score > 0.01 for growth yield in ketoconazole show enrichment in regulation of biological and cellular processes.

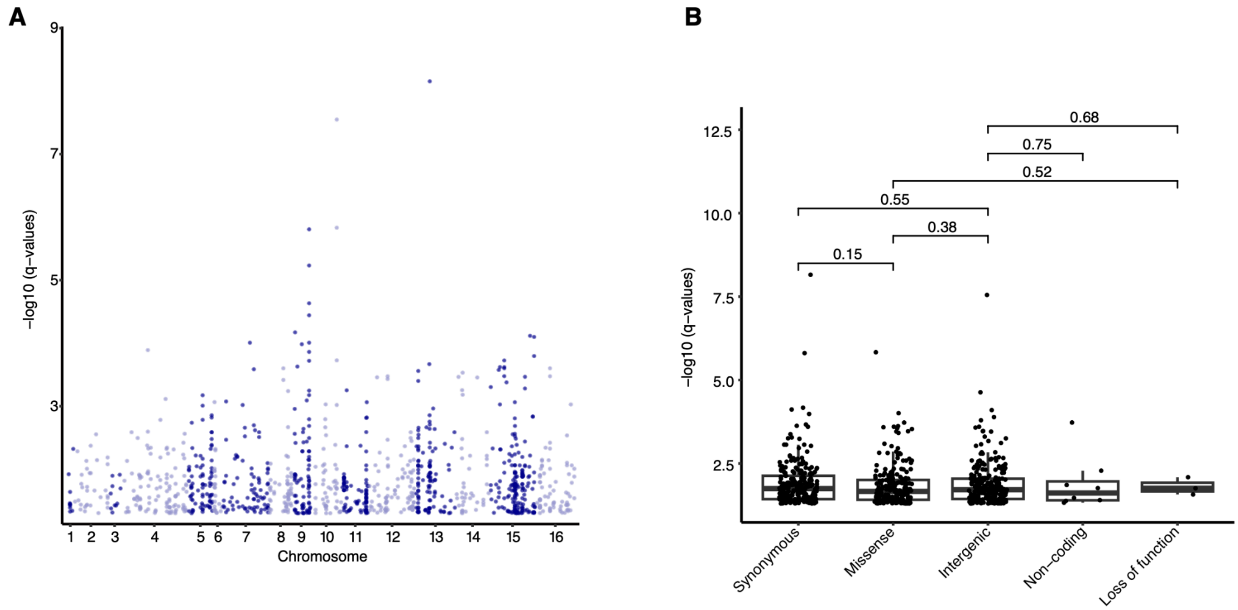

Appendix Figure S11 | Multiple phenotypes correction for GWAS using FDR correction. A, Genome-wide distribution of significant GWAS hits for the global phenome set, corrected for all phenotypes using FDR corrections. **B,** The strength of the associations of GWAS hits does not show any significant differences among various mutation categories after the FDR corrections using all phenotypes.

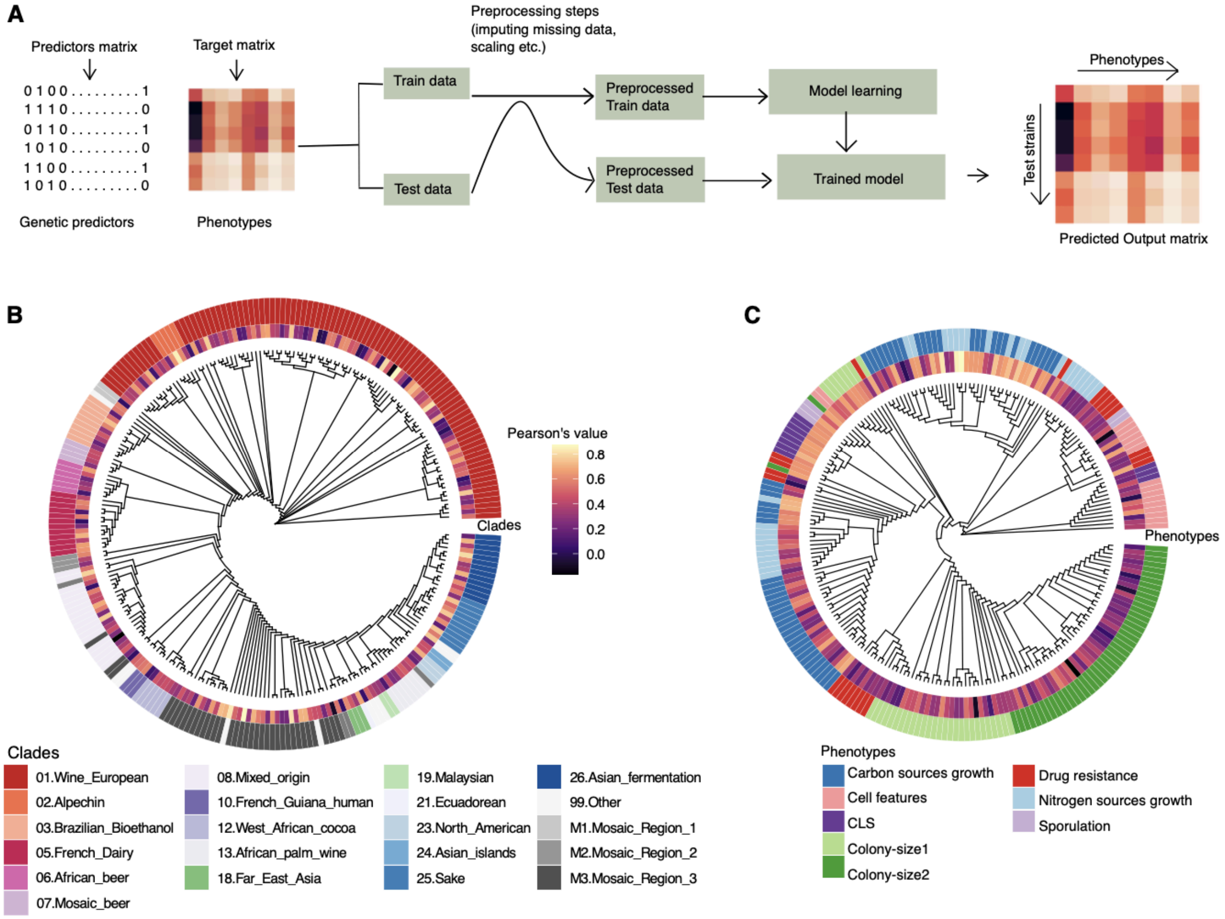

Appendix Figure S12 | Multi-target prediction. A, Schematic of the multi-dimensional target depicting both the input predictor and output phenotype matrices. The samples are represented as rows with features (PA, P(AF) scores, and LOF) and phenotypes as columns. **B,** Prediction accuracy (internal circle) across the phylogenetic tree using pangenome (PA) as the predictor and multitask LASSO as the model. **C,** Prediction accuracy (internal circle) among phenotype classes along the tree based on phenotypic correlations.

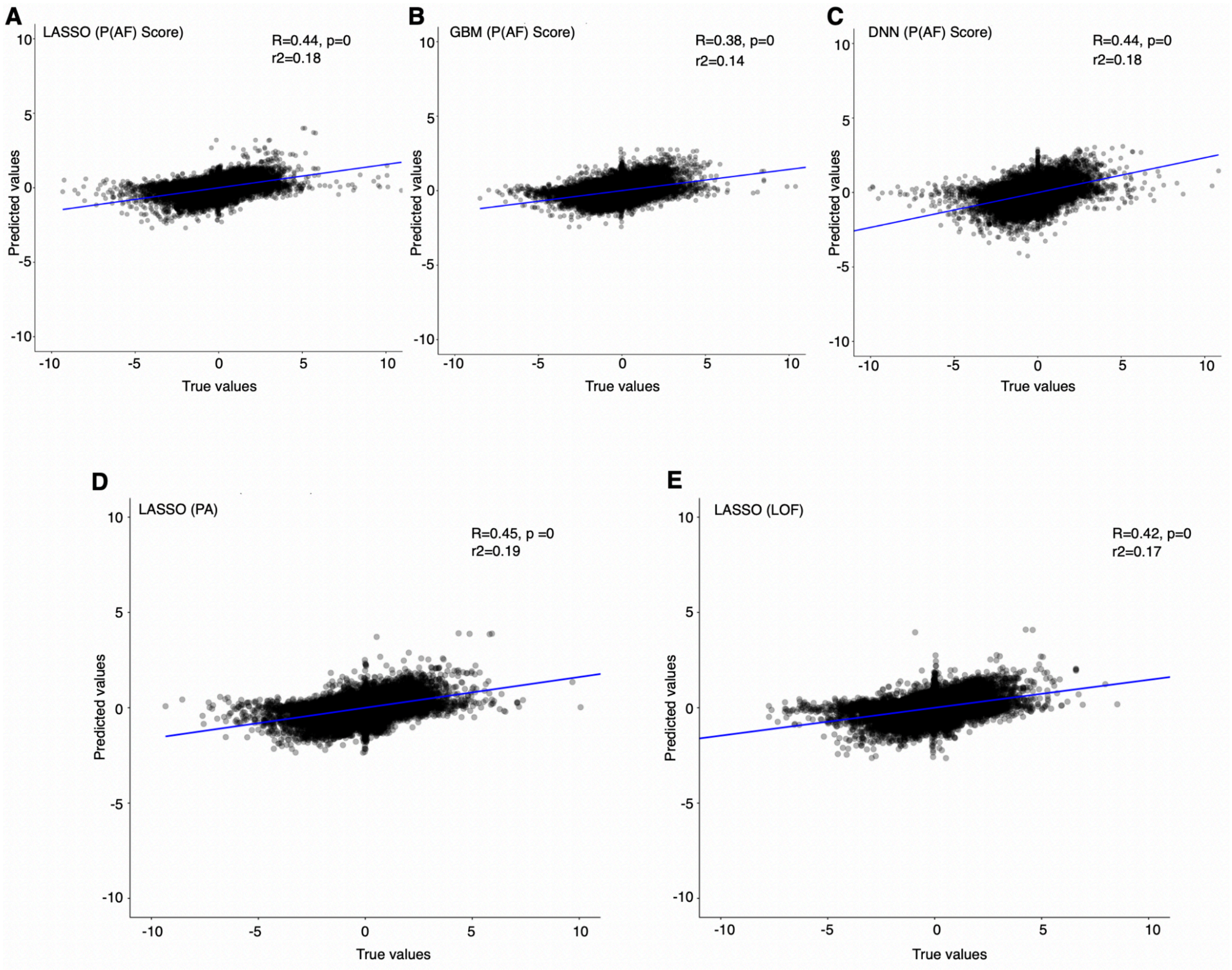

Appendix Figure S13 | Entire phenome predictions. The prediction results measured as the Pearson's correlation coefficient between the true and the predicted values and r2 scores for the entire phenome for the test set are shown for different methods and predictors. **A-C** prediction results for the entire phenome using different methods and (P(AF) scores) as input predictors. The prediction results from linear (LASSO) and non-linear (Neural networks) methods gave very similar results and were slightly better compared to the results from gradient boosting trees. **A, D-E** comparison between the prediction results while using different input predictors with multitask LASSO. All predictors gave similar predictions with Pearson's coefficient ~0.4 and r2 score ~0.18.

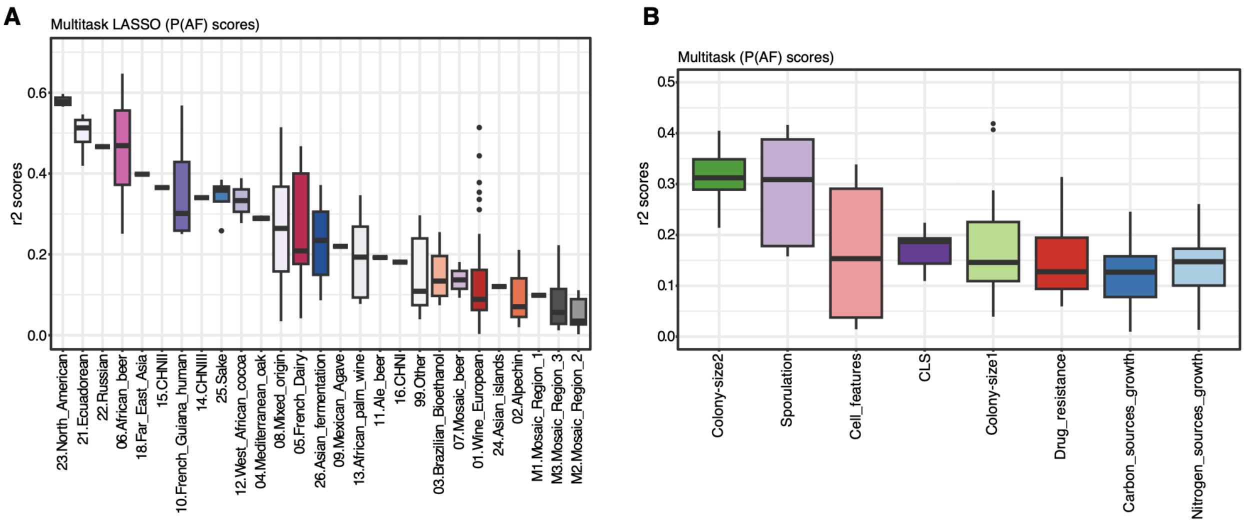

Appendix Figure S14 | Phenome prediction by clades and phenotype classes. The prediction results for the entire phenome with P(AF) scores as predictors and LASSO as prediction method are shown. **A,** The prediction accuracy for all phenotypes with test strains divided according to the clades they belong to shows a large variation ranging from ~0.6 for North American strains to ~0.05 for the strains belonging to mosaic clades. The accuracy remained independent of the size of the clades. **B,** The prediction results show significant variation among different phenotypic classes ranging from 0.3 for colony-size2 consisting of phenotypes measured in stress conditions to 0.15 for growth measured in nitrogen and carbon nutrients.

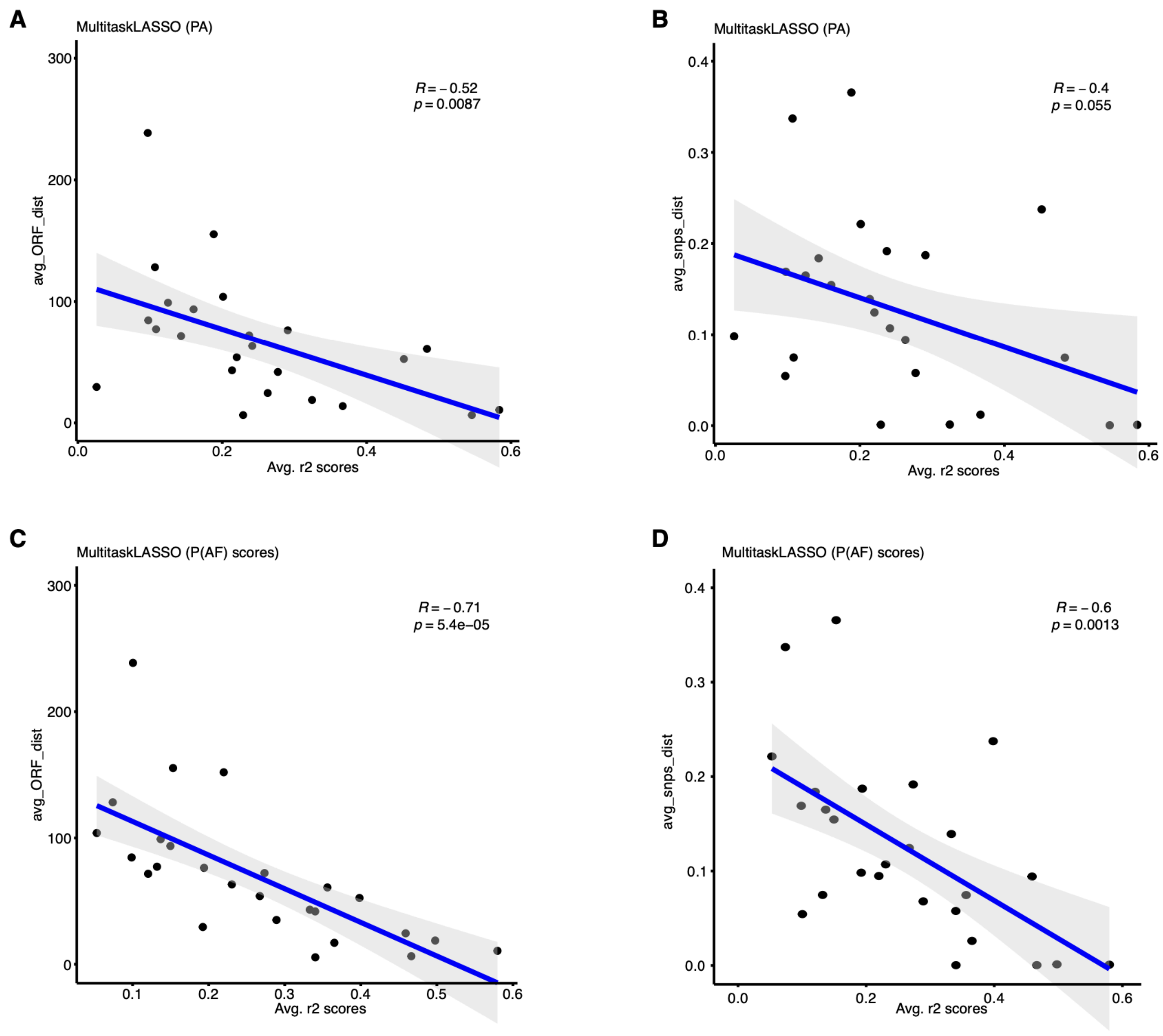

Appendix Figure S15 | Prediction accuracy vs genetic distances. The average prediction accuracies per clade from multitask LASSO vs the average intra-clade genetic variation among the strains measured as the number of ORFs differences (panels (A) and (C)) and the percentage of SNPs differences (panels (B) and (D)) between each pair of strains. In all cases, a significant anticorrelation is observed between the prediction accuracies and intra-clade genetic variation suggesting genetically closer strains phenome is easier to predict compared to distant strains.

Feature selection method	Time (s)	R2 scores	Num. features
Lasso grid	57.5	0.35	206
Lasso random	897.2	0.31	117
Lasso bayes	3042.9	0.13	119
Hi-Lasso	2189.7	0.14	50

Appendix Table S1. Comparing feature selection methods. Comparing different feature selection methods using CLS CR day 21 as the test phenotype and PA as predictor. LASSO grid selection was established as the most efficient method with the highest prediction accuracy.

References

Liu G, Yong MYJ, Yurieva M, Srinivasan KG, Liu J, Lim JSY, Poidinger M, Wright GD, Zolezzi F, Choi H, *et al* (2015) Gene Essentiality Is a Quantitative Property Linked to Cellular Evolvability. *Cell* 163: 1388–1399

Peter J, De Chiara M, Friedrich A, Yue J-X, Pflieger D, Bergström A, Sigwalt A, Barre B, Freel K, Llored A, *et al* (2018) Genome evolution across 1,011 Saccharomyces cerevisiae isolates. *Nature* 556: 339–344

Wagih O, Galardini M, Busby BP, Memon D, Typas A & Beltrao P (2018) A resource of variant effect predictions of single nucleotide variants in model organisms. *Mol Syst Biol* 14: e8430

Whalen S, Schreiber J, Noble WS & Pollard KS (2022) Navigating the pitfalls of applying machine learning in genomics. *Nat Rev Genet* 23: 169–181
