# Supplementary material for: Predicting natural variation in the yeast phenotypic landscape with machine learning
Source: Mol Syst Biol. 2025 Sep 1;21(11):1466–89. doi: 10.1038/s44320-025-00136-y (PMC12583546; doi:10.1038/s44320-025-00136-y)
Supplement: Supplementary file 13 — Expanded View Figures [file 44320_2025_136_MOESM13_ESM.pdf]

Expanded View Figures

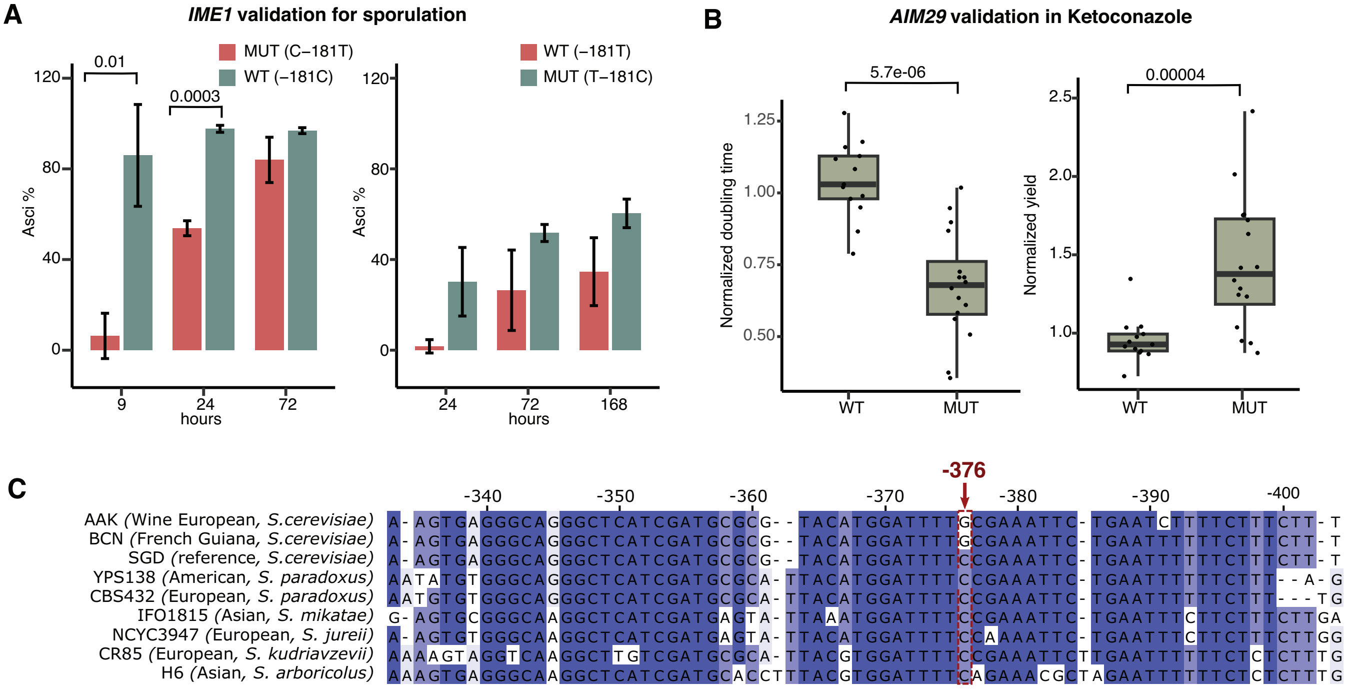

Figure EV1. Highly associated variants.

Panels (**A**, **B**) show experimental validation of the GWAS SNPs in *IME1* promoter and *AIM29* gene respectively. (**A**), Percentage of asci production for two genetic-backgrounds: in the high sportulator strain YPS128 strain (left panel) the native promoter (WT -181C) was edited to the poor sporulating variant (MUT C-181T), resulting in significant difference in asci production at 9 and 24 h (*t* test, error bars indicate standard deviation across 3 replicates). In the poor sporulating strain YJS5833_CIA the native promoter (WT -181T) was edited to the high sporulating variant (MUT T-181C), resulting only in a mild increase in sporulation efficiency, underlying how the single variant effect is shaped by interactions with the genomic background (data replotted from (De Chiara et al, 2022)). (**B**) Growth rate and growth yield in ketoconazole (0.0075 µg/ml) showed significant differences between the BY4743 WT *AIM29* allele (13 replicates) edited for the synonymous variant (MUT C159T, His53His) (16 replicates, Wilcoxon test), data from https://github.com/SakshiKhaiwal/CEFIPRA. The median (50th percentile) of the data is indicated by the black horizontal line in the boxplots, the lower and upper bound of the box indicates the 25th (Q1) and 75th (Q3) percentile, respectively. The whiskers (vertical lines) extends to the smallest and largest value within 1.5* interquartile range (Q3-Q1) and the points beyond the ends of the line are potential outliers. (**C**) Mutation in the promoter region of *SKN7* associated to ketoconazole resistance map within a highly conserved non-coding region of the *Saccharomyces* genus.

© The Author(s)

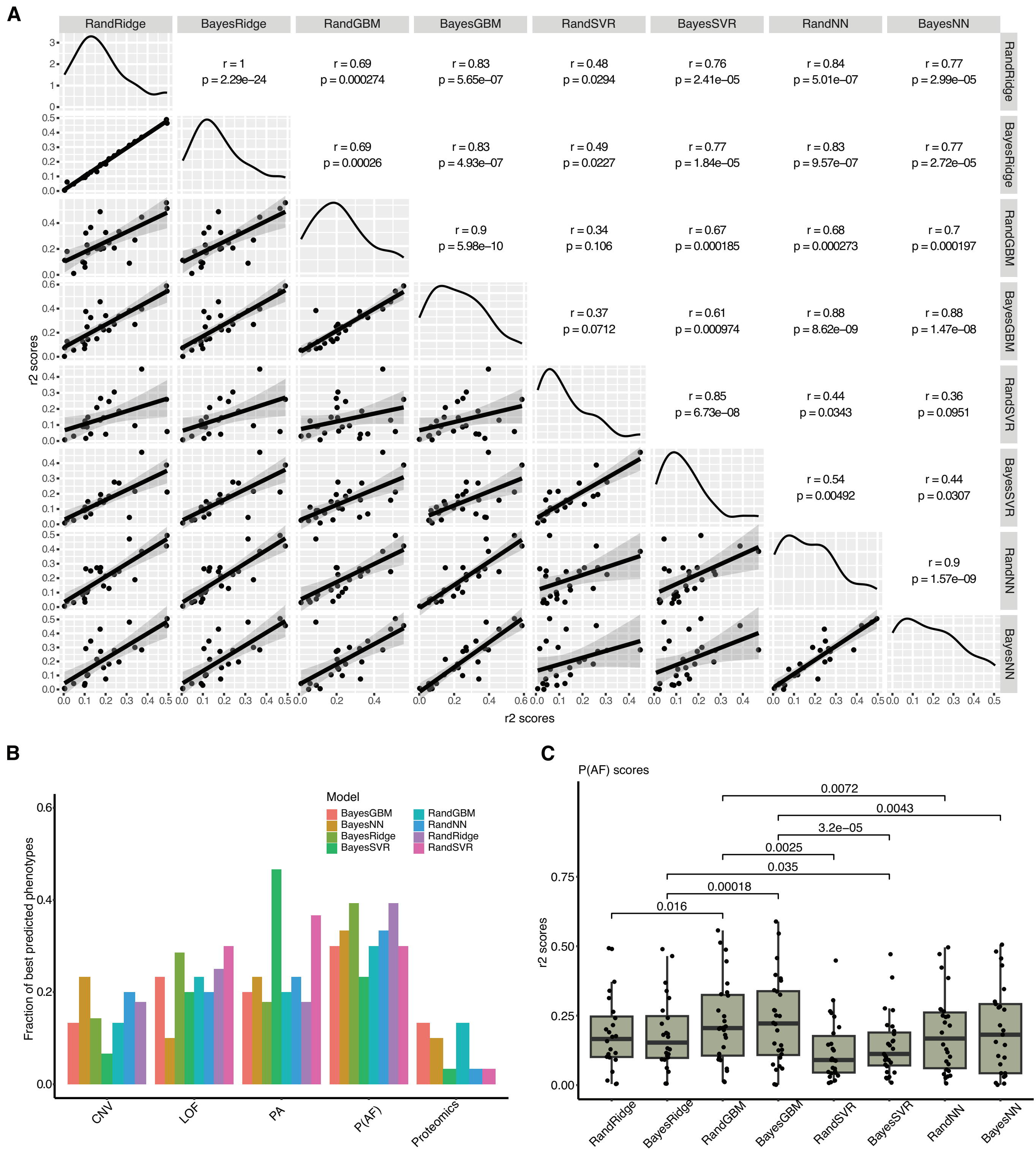
A
RandRidge
BayesRidge
RandGBM
BayesGBM
RandSVR
BayesSVR
RandNN
BayesNN
r = 1
p = 2.29e–24
r = 0.69
p = 0.000274
r = 0.83
p = 5.65e–07
r = 0.48
p = 0.0294
r = 0.76
p = 2.41e–05
r = 0.84
p = 5.01e–07
r = 0.77
p = 2.99e–05
r = 0.69
p = 0.00026
r = 0.83
p = 4.93e–07
r = 0.49
p = 0.0227
r = 0.77
p = 1.84e–05
r = 0.83
p = 9.57e–07
r = 0.77
p = 2.72e–05
r = 0.9
p = 5.98e–10
r = 0.34
p = 0.106
r = 0.67
p = 0.000185
r = 0.68
p = 0.000273
r = 0.7
p = 0.000197
r = 0.37
p = 0.0712
r = 0.61
p = 0.000974
r = 0.88
p = 8.62e–09
r = 0.88
p = 1.47e–08
r = 0.85
p = 6.73e–08
r = 0.44
p = 0.0343
r = 0.36
p = 0.0951
r = 0.54
p = 0.00492
r = 0.44
p = 0.0307
r = 0.9
p = 1.57e–09
r2 scores
r2 scores
B
Model
BayesGBM
BayesNN
BayesRidge
BayesSVR
RandGBM
RandNN
RandRidge
RandSVR
Fraction of best predicted phenotypes
CNV
LOF
PA
P(AF)
Proteomics
C
P(AF) scores
0.0072
0.0043
3.2e–05
0.0025
0.035
0.00018
0.016
r2 scores
RandRidge
BayesRidge
RandGBM
BayesGBM
RandSVR
BayesSVR
RandNN
BayesNN

 © The Author(s)

Figure EV2. Benchmarking ML models on 30 test phenotypes.

(**A**) Pairwise Pearson's correlation comparisons between the prediction accuracies from 8 ML strategies benchmarked using a subset of 30 phenotypes. The two hyperparameter optimization strategies, Bayesian (Bayes) and random (Rand), showed high correlation for each ML model. Moreover, Bayesian GBM showed higher similarity with both ridge and neural networks (NN) compared to random GBM, while SVR showed the highest deviation in the predictions compared to the rest of the models. (**B**) Fractions of phenotypes best predicted (calculated as the number of phenotypes that are best predicted by a given predictor compared to the rest out of all 30 tested phenotypes) with each predictor across 8 methods for the 30 phenotypes test set shows PA and P(AF) scores to be the top predictors. However, each predictor can predict a significant fraction of phenotypes. (**C**) Comparing the distributions of r^2 scores for the 30 phenotypes based on P(AF) scores showed GBM to be the best and SVR to be the worst prediction model. Significant differences between different methods that use the same hyperoptimization technique are reported (paired Wilcoxon test). The median (50th percentile) of the data is indicated by the black horizontal line in the boxplots, the lower and upper bound of the box indicates the 25th (Q1) and 75th (Q3) percentile respectively. The whiskers (vertical lines) extends to the smallest and largest value within 1.5* interquartile range (Q3-Q1) and the points beyond the ends of the line are potential outliers. Source data are available online for this figure.

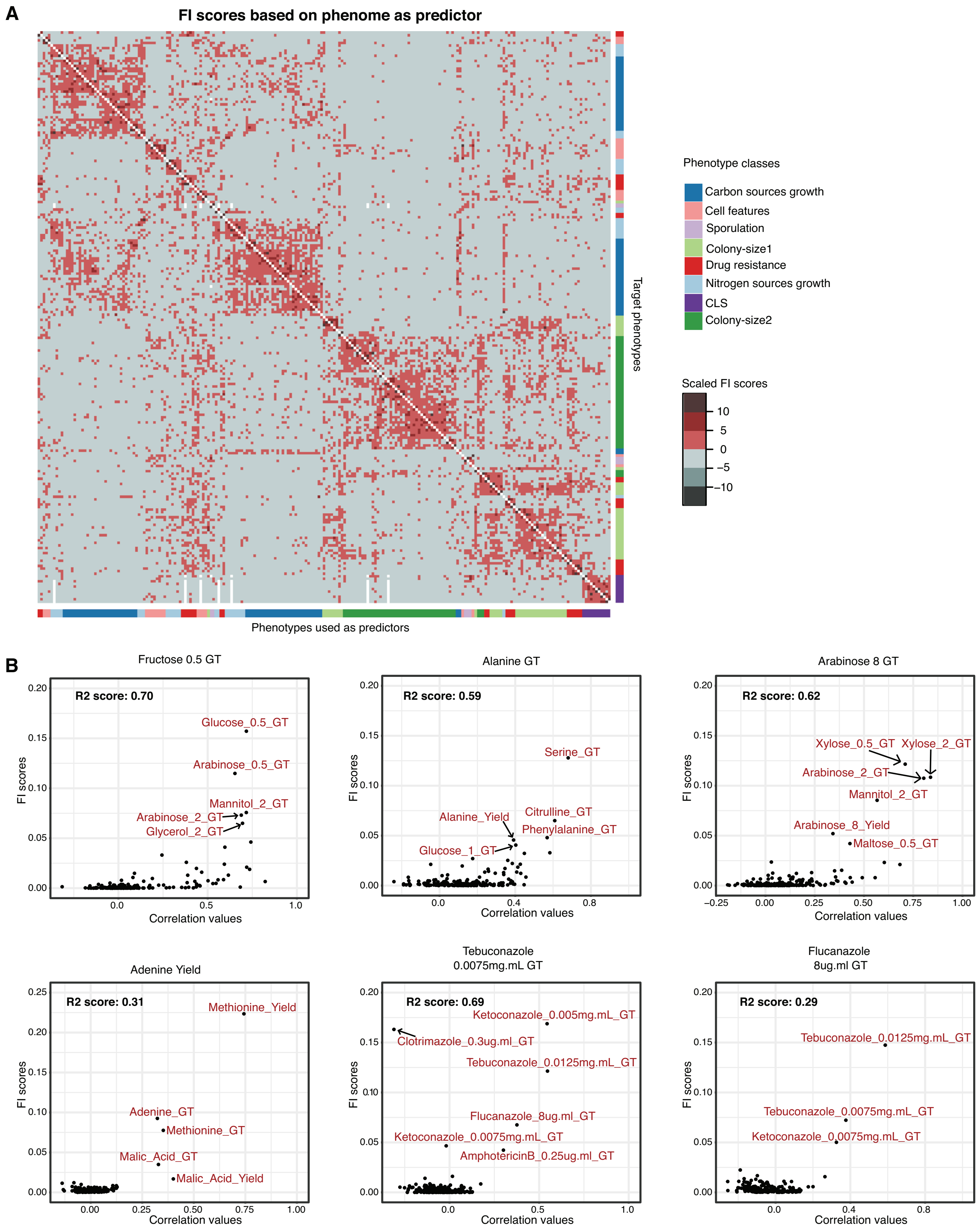
A
FI scores based on phenome as predictor
Target phenotypes
Phenotypes used as predictors
Phenotype classes
Carbon sources growth
Cell features
Sporulation
Colony-size1
Drug resistance
Nitrogen sources growth
CLS
Colony-size2
Scaled FI scores
10
5
0
−5
−10
B
Fructose 0.5 GT
R2 score: 0.70
Glucose_0.5_GT
Arabinose_0.5_GT
Mannitol_2_GT
Arabinose_2_GT
Glycerol_2_GT
Alanine GT
R2 score: 0.59
Serine_GT
Alanine_Yield
Citrulline_GT
Phenylalanine_GT
Glucose_1_GT
Arabinose 8 GT
R2 score: 0.62
Xylose_0.5_GT
Xylose_2_GT
Arabinose_2_GT
Mannitol_2_GT
Arabinose_8_Yield
Maltose_0.5_GT
Adenine Yield
R2 score: 0.31
Methionine_Yield
Adenine_GT
Methionine_GT
Malic_Acid_GT
Malic_Acid_Yield
Tebuconazole 0.0075mg.mL GT
R2 score: 0.69
Ketoconazole_0.005mg.mL_GT
Clotrimazole_0.3ug.ml_GT
Tebuconazole_0.0125mg.mL_GT
Flucanazole_8ug.ml_GT
Ketoconazole_0.0075mg.mL_GT
AmphotericinB_0.25ug.ml_GT
Flucanazole 8ug.ml GT
R2 score: 0.29
Tebuconazole_0.0125mg.mL_GT
Tebuconazole_0.0075mg.mL_GT
Ketoconazole_0.0075mg.mL_GT
FI scores
Correlation values

 © The Author(s)

Figure EV3. Phenotype-based FI scores over phenotype correlations.

(**A**) FI scores for all the phenotypes used as predictors for individual target phenotypes clustered according to phenotype correlations. This heat map shows that larger amounts of information are being shared between similar conditions and traits. (**B**) Examples showing that traits measured in similar conditions are more informative for prediction of growth rate or yield, than traits measured in identical conditions. The *x* axis shows the correlation values between the predicted phenotype (shown on top) and the rest, used as predictors, while the *y* axis depicts the feature importance scores. The r^2 scores from predictions are reported inside each box, while the top feature importance scores are highlighted in red.

© The Author(s)

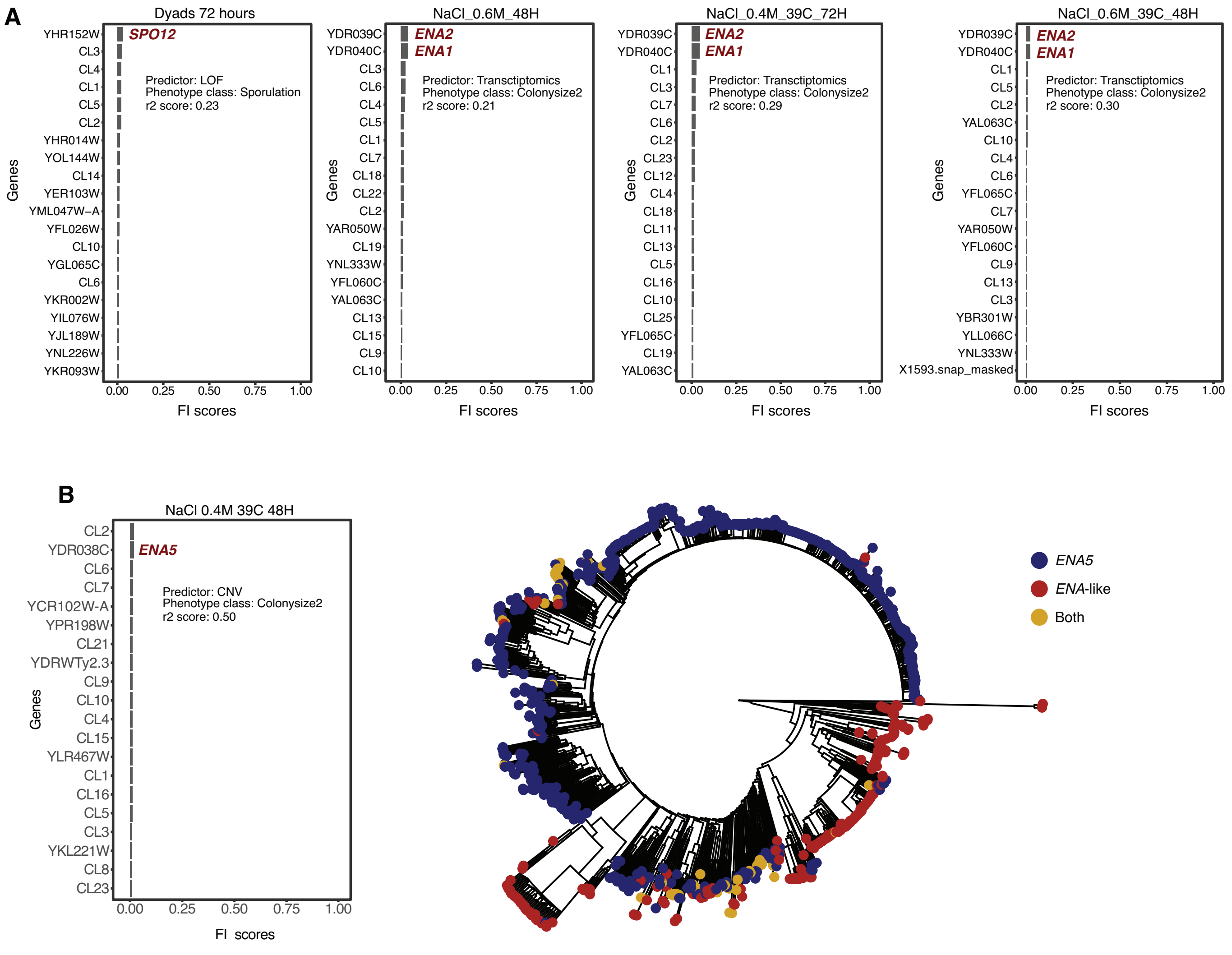

Figure EV4. ML identifies functionally relevant features impacting phenotypes.

(**A**) *SPO12* and *ENA* genes are consistently the top predictor across different time points and concentrations in Dyads production and growth in NaCl respectively. (**B**) *ENA5* (De Chiara et al, 2022; Peter et al, 2018; D'Angiolo et al, 2023; Galardini et al, 2019) gene (representative of the reference-type ENA, which includes *ENA1, ENA2, ENA5* and *ENA6* in the *S. cerevisiae* pangenome (Peter et al, 2018)) is the top gene-predictor for growth measured in NaCl using copy number variation with population structure as the predictor. Population structure features also emerge as top FI scores (depicted by "CL"). This is consistent with the two major ENA alleles, reference-type and *ENA*-like annotated as VAR217 in the *S. cerevisiae* pangenome (Peter et al, 2018) (90% similarity to *ENA* reference), being clade-specific as illustrated by their distribution along the phylogenetic tree (phylogenetic tree, right panel). The phylogenetic tree was drawn and labeled using the Newick file from the 1,011 *S. cerevisiae* collection (Peter et al, 2018).

© The Author(s)
